# Resuscitation of preterm infants in the Philippines: a national survey of resources and practice

**DOI:** 10.1136/archdischild-2019-316951

**Published:** 2019-06-14

**Authors:** Dean Hayden, Maria Esterlita Villanueva-Uy, Maria Katrina Mendoza, Dominic Wilkinson

**Affiliations:** 1 Oxford Uehiro Centre for Practical Ethics, University of Oxford, Oxford, UK; 2 Faculty of Medicine, Nursing and Health Sciences, Monash University, Melbourne, Australia; 3 Institute of Child Health and Human Development, National Institute of Health, Manila, Philippines; 4 Kangaroo Mother Care Foundation, Manila, Philippines; 5 John Radcliffe Hospital, Oxford, UK

**Keywords:** neonatology, resuscitation, intensive care, paediatric practice

## Abstract

**Objective:**

There is a high incidence of preterm birth in low-income and middle-income countries where healthcare resources are often limited and may influence decision making. We aimed to explore the interplay between resource limitations and resuscitation practices for extremely preterm infants (EPIs) in neonatal intensive care units (NICUs) across the Philippines.

**Methods:**

We conducted a national survey of NICUs in the Philippines. Institutions were classified according to sector (private/public), region and level. Respondents were asked about unit capacity, availability of ventilators and surfactant, resuscitation practices and estimated survival rates for EPIs of different gestational ages.

**Results:**

Respondents from 103/228 hospitals completed the survey (response rate 45%). Public hospitals reported more commonly experiencing shortages of ventilators than private hospitals (85%vs23%, p<0.001). Surfactant was more likely to be available in city hospitals than regional/district hospitals (p<0.05) and in hospitals classified as Level III/IV than I/II (p<0.05). The financial capacity of parents was a major factor influencing treatment options. Survival rates for EPIs were estimated to be higher in private than public institutions. Resuscitation practice varied; active treatment was generally considered optional for EPIs from 25 weeks’ gestation and usually provided after 27–28 weeks’ gestation.

**Conclusion:**

Our survey revealed considerable disparities in NICU resource availability between different types of hospitals in the Philippines. Variation was observed between hospitals as to when resuscitation would be provided for EPIs. National guidelines may generate greater consistency of care yet would need to reflect the variable context for decisions in the Philippines.

**What is already known on this topic?**

- Many professional organisations and societies have developed management guidelines to support clinicians facing decisions about offering or withholding resuscitation for extremely preterm infants. There are no available guidelines that provide specific advice to clinicians in low-income or middle-income country while incorporating considerations of resource constraints. Studies from these settings have shown significant variation in resuscitation practices between and within countries.

**What this study adds?**

- This is the first study to assess both availability of neonatal intensive care unit resources and resuscitation practices of preterm infants across an entire low-income or middle-income country.

## Introduction

In many countries, professional organisations have developed guidelines for the perinatal care of extremely preterm infants (EPIs), including when resuscitation should or should not be provided. Most existing guidelines indicate thresholds based on the infant’s gestational age (GA).^1 2^ A *lower threshold* marks the gestation before which treatment will not usually be provided. An *upper threshold* marks the point after which treatment is considered mandatory. Between these ages lies a ‘grey zone’, where active treatment may or may not be provided, and parents’ wishes are important.

Published guidelines stem from high-income countries or international bodies.^3–5^ However, the vast majority of preterm births globally occur in low-income and middle-income countries (LMICs), where resource limits can affect the provision of medical care.^6–10^ To our knowledge, there are no published national guidelines for clinicians resuscitating preterm infants in these settings. Several studies have examined practice in individual hospitals or regions in LMICs, indicating variation between and within countries in the GA and birth weight thresholds used for resuscitation.^11–18^

The aim of this study was to survey neonatologists across an LMIC about resuscitation decisions for EPIs. We aimed to assess whether resuscitation practices varied between sectors of the health system and to identify the influence of resource limitations.

The Philippines is an archipelagic country with a population of 105 million and a gross domestic product (GDP)/capita of US$2989.^19^ Worldwide, it ranks eighth highest in number of preterm births (350 000/year).^6 20^ The average neonatal mortality rate is estimated to be 14 per 1000 live births, though there is wide regional variation (it is lower in urban areas but >20/1000 live births in some provinces).^21 22^ As in many other LMICs, for both private and public facilities, parents are required to pay out of pocket for the care of their infant.^23^

## Methods

### Participants

Neonatologists working in neonatal intensive care units (NICU) in the Philippines were contacted through a database provided by the Philippine Pediatric Society. For each hospital, a single neonatologist was identified to respond on their institution’s behalf. In some cases, one clinician was asked to complete the survey more than once, on behalf of multiple hospitals, as they were the only neonatologists working at those institutions. Up to three reminder emails and a single text message were sent to non-responders.

### Evaluation instrument

A 34-item SurveyMonkey questionnaire was developed. The survey was written and conducted in English, an official language of the Philippines.^24 25^ Participation was voluntary, and responses were anonymous. The survey was conducted between January and March 2018.

The survey was structured in three main parts. The first requested information on the characteristics of the hospital including numbers of overall and preterm births. The second focused on availability of resources including number of beds, mechanical ventilators and surfactant. The third requested information about hospital policies and practice relating to resuscitation of EPIs. Respondents were asked how often resuscitation would be provided for infants born at a given gestation.

Questions consisted of Likert scale responses, yes/no, multiple choices and open-ended questions. For questions asking the frequencies of particular occurrences, ‘never’ and ‘rarely’ responses were grouped into a single category, as were ‘often’ and ‘almost always’ in our analysis. Clinicians were also asked about factors influencing decision making.^14^

Lastly, the survey asked for sociodemographic information from respondents including level of experience, religion and level of religiosity.

### Statistical analysis

Data were analysed using the Statistical Package for Social Sciences (SPSS) v25.0.^26^ Bivariate analyses to assess association between categorical outcomes were performed using Fisher’s exact test. Mann-Whitney U and Kruskal-Wallis tests were used for assessing differences between continuous variables. We analysed separately survival estimates for Level III/IV hospitals, as these would have most experience of caring for EPIs.

We assessed the frequency by which each institution would initiate resuscitation for infants of a given GA. We performed a post hoc analysis using Fisher’s exact test to assess if responses differed between different types of institutions at either 23/24 weeks’ gestation or 27/28 weeks’ gestation.

## Results

### Participants

Of the 228 hospitals providing neonatal care in the Philippines, we received 103 responses, yielding an overall response rate of 45%. Responses were submitted by 83 different neonatologists (14 entered data for more than one hospital). Most responses were from city hospitals, and approximately two-thirds were from private hospitals (table 1). The majority of respondents (75%) had more than 10 years’ experience of working in NICU.

**Table 1:** Hospital and respondent characteristics

	No. (%)
**Hospital classification (n=103)**	
Administrative sector	
Public/government	34 (33)
Private	69 (67)
Region*	
City	81 (79)
Provincial	17 (16)
District	5 (5)
**Level**†	
Level I	6 (6)
Level II	28 (27)
Level III	56 (54)
Level IV	10 (10)
Unknown/unclassified	3 (3)
**Respondent characteristics (n=83)**	
Professional role	
Consultant	81 (98)
Registrar/fellow	2 (2)
Gender	
Male	21 (25)
Female	62 (75)
**Years working in NICU**‡	
1–5 years	12 (15)
6–10 years	8 (10)
11–15 years	14 (17)
16–20 years	27 (33)
>20 years	20 (24)
Religious belief	
Yes	83 (100)
Atheist/agnostic	0
**Religious denomination**§	
Christianity – Catholic	68 (82)
Christianity – Evangelical	6 (7)
Christianity – Born Again Christians	6 (7)
Christianity – Baptist	2 (2)
Prefer not to say	1 (1)
**Importance of religion**§	
Most important	31 (37)
Very important	50 (60)
Fairly important	2 (2)
Not important	0

*Hospitals in the Philippines fall under different regional administrative units; city hospitals (managed by city governments), ‘district’ and ‘provincial’ hospitals (both managed by the provincial government and the latter providing tertiary care to a greater catchment area).^23^

†Hospitals are classified into four different levels according to the types of facilities available. Level 1 and 2 hospitals are well distributed across the entire country, while higher level hospitals are concentrated in fewer regions with greater population density.^33^

‡Numbers sum up to 81 as two respondents did not answer this question.

§For respondents who indicated that they had a religion. Refers to importance of religion in respondents’ lives.

NICU, neonatal intensive care unit.

### Births and resource availability

Respondents from higher level hospitals reported a larger number of beds and ventilators (online supplementary appendix 1). Compared with private hospitals, public hospitals reported a significantly higher number of births and preterm births, a larger number of beds but a lower number of ventilators per 100 preterm births.

10.1136/archdischild-2019-316951.supp1 Supplementary data

Most hospitals reported experiences of limitations in available ventilators at least some of the time (table 2). While 32% of private hospitals reported being ‘often’ or ‘always’ at full capacity for ventilators, this was reported by 85% of public hospitals (p<0.001).

**Table 2:** Frequency of resource limitations in newborn intensive care: mechanical ventilation and surfactant administration

	Level		P value*	Administration		P value*	Region			P value*
	I/II	III/IV		Public	Private		City	Provincial	District	
Frequency of situations where all mechanical ventilators are in use and at least one other infant needs mechanical ventilation†										
Never/rarely	5 (15)	17 (26)	0.04	2 (6)	21 (30)	<0.001	22 (28)	1 (5.9)	0	0.003
Some of the time	15 (44)	13 (20)		3 (9)	26 (38)		26 (33)	1 (5.9)	2 (40)	
Often/almost always	14 (41)	35 (54)		28 (85)	22 (32)		32 (40)	15 (88.2)	3 (60)	
Availability of surfactant for preterm infants with respiratory distress†										
Never	3 (9)	5 (8)	0.02	6 (19)	2 (3)	0.06	3 (4)	4 (27)	1 (20)	0.02
Some of the time	6 (18)	6 (10)		3 (10)	10 (15)		12 (15)	1 (7)	0	
Only if parents are able to pay‡	19 (56)	21 (34)		13 (42)	29 (43)		32 (41)	6 (40.0)	4 (80)	
Always	6 (18)	30 (48)		9 (29)	27 (40)		32 (41)	4 (27)	0	

*Fisher’s exact test. P values represent differences in overall distribution of responses between different hospitals types.

†Rarely=less than once per year, some of the time=more than once per year but less than once per month, often=more than once per month but less than once per week and almost always=more than once per week.

‡This option included situations where the parents are able to find a charity to cover the costs of care.

Forty-one per cent of hospitals reported that surfactant for respiratory distress syndrome was available only if parents or a charity were able to pay for it. Surfactant was more likely to be ‘always’ available in higher level hospitals (p<0.05) and more often available in city hospitals than provincial/district hospitals (p<0.05).

The most common responses to ventilator shortages were for the family to rent a ventilator, to attempt hand-ventilation, or to transfer the infant to another facility (table 3). Most hospitals reported no specific limitations on which preterm infants receive surfactant. Of the hospitals that did report a limiting factor, most cited the family’s financial capacity.

**Table 3:** Responses of hospitals to limitations in availability of mechanical ventilators or surfactant

	No. (%)*
**Course of action if all ventilators are in use and another infant requires mechanical ventilation (n=92)**	
The family will hire a ventilator from a rental company	52 (57)
Hand ventilation is attempted if there are individuals able to do so	40 (44)
The neonate is transferred to a facility with an available ventilator	37 (40)
Babies who are currently on the ventilator and who are on low ventilation settings are taken off support in the hope that they won’t need it	12 (13)
The hospital will hire a ventilator from a third party or source one from another unit	12 (13)
CPAP is attempted (either nasal or ET)	4 (4)
New babies who need treatment are kept comfortable and die	1 (1)
**Rules or limitations placed on which infants are able to receive surfactant: (n=65)**	
No rules or limitations placed	29 (45)
Financial capacity of family	23 (35)
The infant must fall within a particular GA range	6 (9)
There is a maximum number of doses due to cost	4 (6)
Availability of medication	4 (6)
Availability of a mechanical ventilator	1 (2)
The infant must be of a minimum birth weight	1 (2)

*Respondents could select more than one answer. Percentages reflect the proportion of respondents who selected a particular answer, therefore percentages do not total 100.

CPAP, continuous positive airway pressure; ET, endotracheal tube; GA, gestational age.

### Costs of care

The median reported costs to parents for different services are shown in online supplementary appendix 2. The costs of daily NICU care, ventilator rental and surfactant administration were significantly higher in private hospitals.

10.1136/archdischild-2019-316951.supp2 Supplementary data

### Survival rates

**Figure 1:**
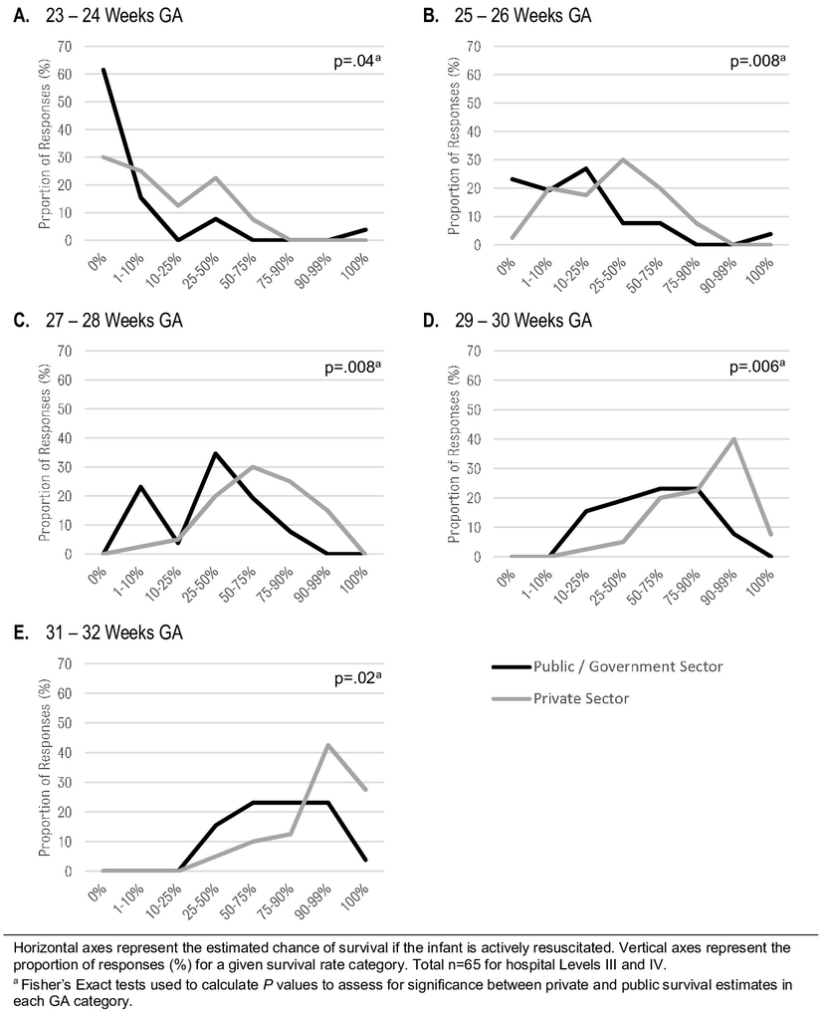
Estimated survival rates (if actively treated) at different GA categories in Level III and IV hospitals. GA, gestational age.

The estimated rate of survival for preterm infants increased with GA (figure 1), though there was wide variation in the estimates. Respondents consistently estimated higher survival rates for preterm infants cared for in private institutions than in public hospitals. City hospitals reported higher estimated survival than district/provincial hospitals at all gestations except at 23–24 weeks’ GA (online supplementary appendix 3).

10.1136/archdischild-2019-316951.supp3 Supplementary data

### Initiating and limiting resuscitation for EPIs

**Figure 2:**
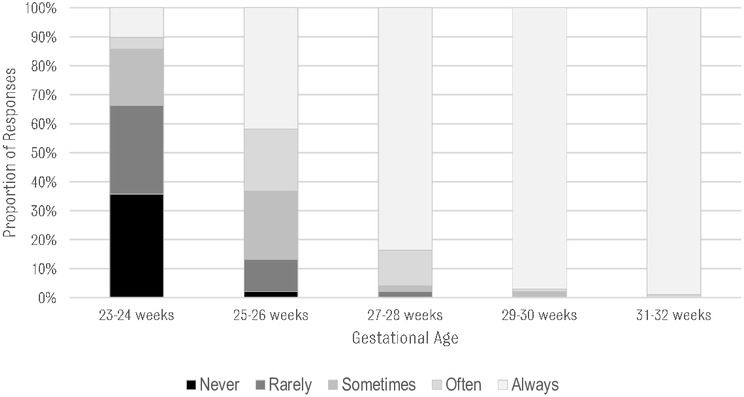
Frequency of initiating resuscitation for a given gestational age.

A majority of respondents reported using the American Academy of Pediatrics Neonatal Resuscitation Program (NRP) guidelines (online supplementary appendix 4).^27^ Fourteen hospitals reported using a local guideline developed by their department or institution. When asked about resuscitation at 23–24 weeks’ GA, 14% indicated that at their institution infants would ‘always’ or ‘often’ be resuscitated, while 66% indicated that resuscitation would ‘never’ or ‘rarely’ be initiated (figure 2). There was no significant difference between sectors, region or hospital level in the frequency of resuscitating at 23/24 weeks’ gestation (online supplementary appendix 5A). At 25–26 weeks’ gestation, 41% of hospitals would ‘always’ resuscitate, while 21% would ‘often’, and 23% would ‘sometimes’ resuscitate.

10.1136/archdischild-2019-316951.supp4 Supplementary data

10.1136/archdischild-2019-316951.supp5 Supplementary data

At 27–28 weeks’ GA, 84% of respondents indicated that they would always resuscitate (figure 2). The reported reasons for non-resuscitation in this group are listed in online supplementary appendix 5B.

Resuscitation was almost always provided at higher GAs (97% and 99% in the 29–30 and 31–32 weeks’ GA groups, respectively).

The most frequently cited reason influencing clinicians’ decision to limit resuscitation was ‘respecting parents’ wishes’ followed by ‘probability of infant death’ and ‘clinician’s morals’ (online supplementary appendix 6).

10.1136/archdischild-2019-316951.supp6 Supplementary data

Most respondents reported also using birth weight as a threshold for initiating resuscitation if the GA were uncertain (online supplementary appendix 7). The median birth weight threshold reported was 500 g (range of 400–800 g).

10.1136/archdischild-2019-316951.supp7 Supplementary data

No significant associations between willingness to offer resuscitation and participant characteristics were observed.

## Discussion

To our knowledge, this is the first study to report the availability of NICU resources and resuscitation practice for EPIs across multiple hospital sectors in an LMIC. We found large disparities between hospitals across the Philippines in the availability of NICU resources. Furthermore, we found that the financial capacity of parents influenced treatment limitation decisions and played a major role in the response to resource limitations. Our study indicated that resuscitation would generally be considered from 25 weeks’ gestation in the Philippines and would usually be provided from 27 to 28 weeks’ gestation, though, in the absence of national guidance, there was large variation in when resuscitation or non-resuscitation would be considered. We did not observe disparities in resuscitation practices between different types of hospitals; however, our results suggested that outcomes for EPIs may differ between sectors; respondents provided higher survival estimates for EPIs in private versus public hospitals.

### Resource limitations

The number of NICU ventilators per 100 preterm births (a metric we devised for assessing resource capacity) showed striking differences between the private and public sector. The reported differences in availability of neonatal beds, ventilators and surfactant were generally consistent with the intuitive assumption that large, private, urban centres would be better resourced than small, public, rural centres, respectively.

Hospitals reported wide-ranging courses of action taken in the face of resource limitations. When ventilators are at full capacity, the most commonly cited course of action was for the family to rent a ventilator, which may not be possible for poor families. It is possible that in such cases, hospitals resort to other cited responses such as hand ventilation, or attempt to identify a source of charitable funding for the family.^28^ Approximately one-third (35%) of respondents cited financial capacity of the family as a limiting factor in the provision of surfactant. The reported cost of a single dose of surfactant represents approximately 9% of the average per capita annual income in the Philippines.^19^ For some families, incurring the costs associated with extreme prematurity may lead to financial catastrophe.

Previous studies in other LMICs have documented the financial capacity of parents as a limiting factor in resuscitation of their preterm infant. Qualitative studies from neonatal units in India have reported that families’ motivations for withdrawing treatment were often based on costs, and that clinicians would only resuscitate EPIs if the family was willing to pay the entire bill themselves.^16 29^

We found differing estimated survival rates between different hospital types. Rates were significantly lower in public hospitals compared with private hospitals for all GA bands (figure 1). Lower survival rates in public hospitals might relate to the different number and capacity of ventilators reported in our study (tables 2 and 3). Poorer outcomes might also relate to other factors, including staffing levels, experience, rates of nosocomial infection or overcrowding.^25 30^ It might also conceivably relate to different risk factors in women presenting to public institutions (eg, reduced antenatal care and fetal growth restriction relating to maternal malnutrition).^31 32^

### Resuscitation

Our survey identified that there is no single widely adopted guideline in the Philippines for resuscitation of EPIs (online supplementary appendix 4). The most commonly cited guideline, reported by 54% of respondents, was a US guideline. The NRP 7th Edition identifies the grey zone for resuscitation between 22 and 24 weeks GA, and does not take into consideration constraints on resources.^27^ These guidelines may not be easily applicable to the Philippine context, and indeed, local practice seemed to differ from the NRP recommendations. In Philippine hospitals, resuscitation was generally considered to be an option from 25 weeks’ gestation.

Thresholds reported in several other LMICs resemble our findings. Resuscitation is generally only considered beyond 25 weeks GA in South Africa, Lebanon and Malaysia.^12 14 17^ In contrast, studies from elsewhere report thresholds at later GAs: 26 weeks in El Salvador, 28 weeks in India and 31 weeks in Mongolia.^13 15 29^

As expected, in the absence of national guidelines, we found significant variations between institutions. Particularly at lower GA (23–26 weeks), we found a striking degree of heterogeneity, with some centres reporting that they would never or rarely resuscitate, while others reported usually or always providing resuscitation. Practice appeared more consistent for infants on reaching the 29–30 weeks’ GA band. At 27–28 weeks, some centres cited parents’ inability to pay as a reason to withhold treatment, while others indicated that this was based on anticipated poor outcome.

The divergence of practice seen across institutions could be due to differences in resource capacity. However, we did not observe differences in the reported rate of resuscitation between the different hospital types.

The spectrum of approaches (resuscitation offered for some infants at 23 weeks and withheld for some infants at 28 weeks) may represent a wide grey zone in which resuscitation is provided predominantly according to parental wishes. Approximately 2/3 of respondents reported that ‘parents’ wishes’ and ‘financial cost (for family)’ often or always affected decisions to limit resuscitation (online supplementary appendix 6). This contrasts starkly with an earlier survey of clinicians in six Pacific countries. In that study, only 26% of clinicians in Malaysia and 1% of clinicians in Japan reported ‘financial cost (for family)’ to often or always affect decisions to limit resuscitation.^14^ Of concern, our study suggested a higher prevalence of litigation fear influencing resuscitation decisions (29%) compared with other Pacific rim countries.

### Limitations

We had modest response rates, which may affect the generalisability of our results, though the proportion of private and public hospital respondents in our study was close to the nationwide ratio. We had a higher proportion of level III/IV hospitals than the nationwide distribution (approximately 25%), yet many level I/II hospitals lack newborn care services and were not invited to participate in our study.^23^

Each hospital’s response was restricted to the views of one neonatologist responding on its behalf. Respondents may have answered some questions according to their own practice, which in the absence of clear guidelines may differ from their colleagues. We relied on participant’s reporting of outcomes (ie, estimated survival rate), yet such recollections or impressions of outcomes may be inaccurate or biased. We were not able to verify the accuracy of such estimates due to a general lack of evidence on outcomes for EPIs in the Philippines.

## Conclusion

Our study provides valuable insights into the challenges of neonatal care in an LMIC. Philippine paediatricians are endeavouring to provide the same level of care that is available in developed countries; however, resource scarcity and the costs of treatment appear to be critical to decisions in a way that they are not in more well-resourced health systems. National guidelines would potentially generate greater consistency of care for preterm infants. However, they would need to reflect the context of decision making in a LMIC. The Philippine Society of Newborn Medicine is currently in the process of developing a national consensus guideline.

One challenge in establishing guidelines is the need for locally relevant data on the outcomes of treatment. The short-term and long-term outcomes of infants resuscitated at different GAs and birth weights in the Philippines would be highly useful, yet such data are currently lacking.

Finally, the perceived difference in outcome for EPIs between private and public institutions and the large out-of-pocket costs for families (a problem likely shared with other LMICs), points to the need for ethical attention to the structure and extent of funding for children’s healthcare in the Philippines.

10.1136/archdischild-2019-316951.supp8 Supplementary data
