# Supplementary material for: Resuscitation of preterm infants in the Philippines: a national survey of resources and practice
Source: Arch Dis Child Fetal Neonatal Ed. 2019 Jun 14;105(2):209–14. doi: 10.1136/archdischild-2019-316951 (PMC7063403; doi:10.1136/archdischild-2019-316951)
Supplement: Supplementary data [file fetalneonatal-2019-316951supp001.pdf]

Appendix 1

Numbers births and available resources in Philippine hospitals (grouped by level, administration and region)

	Level				Administration		Region			Total
	I (n=6)	II (n=28)	III (n=56)	IV (n=10)	Public (n=34)	Private (n=69)	City (n=81)	Provincial (n=17)	District (n=5)	
**Births (median)**										
Number of births in 2017	1500.0	750.0	1188.0	2000.0	5186.0	718.0[a]	845.0	6213.0	1200.0	1000.0
Number of preterm births in 2017	50.0	60.0	100.0	150.0	296.5	59.5[a]	70.0	463.0	240.0	80.0
Percentage preterm[d]	4.5	8.4	9.3	10.5	9.4	8.3	8.3	8.0	10.0	8.3
**Resources (median)**										
Official number of beds in NICU	4.0	9.0	11.0	10.0[b]	15.0	9.0[a]	10.0	15.0	10.0	10.0
Maximum number of beds in NICU	5.0	10.0	15.0	16.0[c]	27.0	10.0[a]	12.0	25.0	15.0	14.0
Number of ventilators part of the standard NICU setup	1.0	2.0	3.0	5.0[a]	4.0	2.0[a]	3.0	3.0	1.0	3.0
Number of ventilators per 100 preterm births	1.2	3.6	3.7	3.3	0.9	4.3[a]	4.0	0.8	0.4	3.3

Abbreviations: NICU – Neonatal Intensive Care Unit.

[a] P<.001, [b] P=.01, [c] P=.02. Mann-Whitney U tests comparing Public vs Private hospitals. Kruskal-Wallace tests comparing Levels I-IV.

[d] Percentage preterm calculated individually for each hospital as number of preterm births divided by number of births. Values in this row represent medians of these percentages.
