# Supplementary material for: Resuscitation of preterm infants in the Philippines: a national survey of resources and practice
Source: Arch Dis Child Fetal Neonatal Ed. 2019 Jun 14;105(2):209–14. doi: 10.1136/archdischild-2019-316951 (PMC7063403; doi:10.1136/archdischild-2019-316951)
Supplement: Supplementary data [file fetalneonatal-2019-316951supp002.pdf]

Appendix 2

Costs of neonatal care charged to parents (median): Private vs Public

`	Overall	Public	Private	*P* Value[a]
Cost of general NICU services (per day)	₱4,500 ($87)	₱2,000 ($39)	₱5,000 ($96)	.007
Hiring a ventilator (per day)	₱2,000 ($39)	₱1,000 ($19)	₱2,500 ($48)	.001
Surfactant administration (per dose)	₱16,550 ($319)	₱12,500 ($241)	₱20,000 ($386)	<.001

Abbreviations: NICU – Neonatal Intensive Care Unit

[a] Using Mann-Whitney U test for comparison. Prices expressed as mean values in Philippine Piso (PHP) currency and US Dollars (USD). Prices were converted into USD using the monthly average exchange rate for February 2018.[1]

The 2017 Gross National Income (GNI) per capita of the Philippines was $3660 (USD) or ₱184,420 (PHP).[2]
