# Supplementary material for: Resuscitation of preterm infants in the Philippines: a national survey of resources and practice
Source: Arch Dis Child Fetal Neonatal Ed. 2019 Jun 14;105(2):209–14. doi: 10.1136/archdischild-2019-316951 (PMC7063403; doi:10.1136/archdischild-2019-316951)
Supplement: Supplementary data [file fetalneonatal-2019-316951supp003.pdf]

Appendix 3

Comparing estimated survival rates between city hospitals and district/provincial hospitals for different gestational age categories

A. 23 – 24 Weeks GA

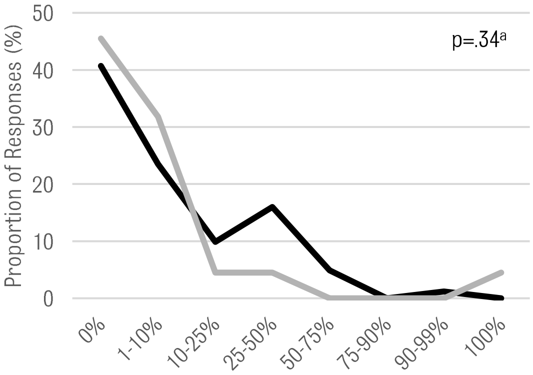

B. 25 – 26 Weeks GA

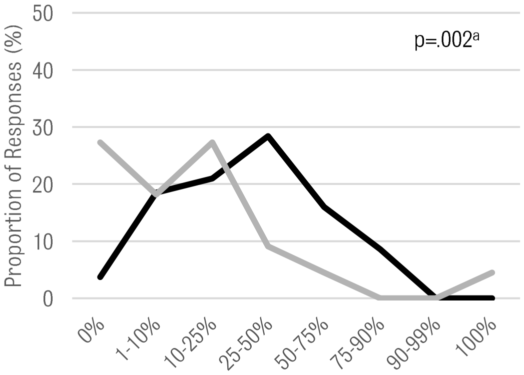

C. 27 – 28 Weeks GA

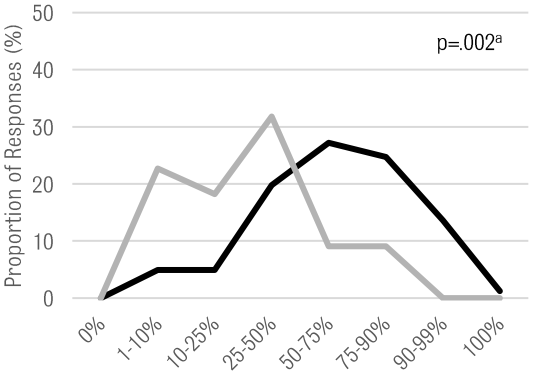

D. 29 – 30 Weeks GA

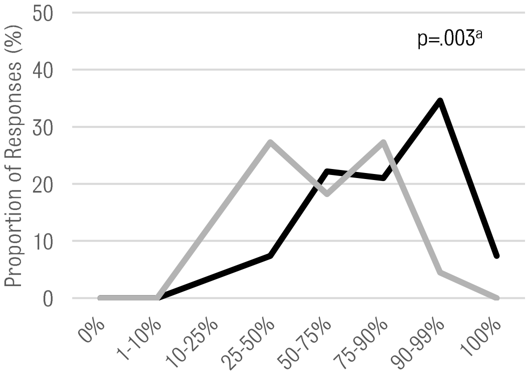

E. 31 – 32 Weeks GA

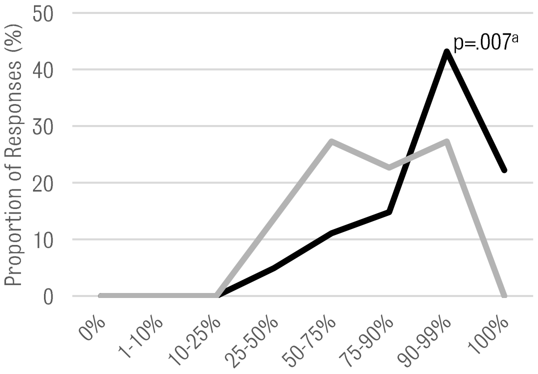

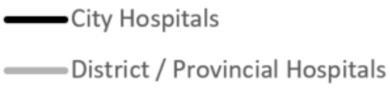

Horizontal axes represent the estimated chance of survival if the infant is actively resuscitated. Vertical axes represent the proportion of responses (%) for a given survival rate category.

[a] Fisher's Exact tests used to calculate p-values to assess for significance between City and District/Provincial survival estimates in each GA category.
