# Supplementary material for: Resuscitation of preterm infants in the Philippines: a national survey of resources and practice
Source: Arch Dis Child Fetal Neonatal Ed. 2019 Jun 14;105(2):209–14. doi: 10.1136/archdischild-2019-316951 (PMC7063403; doi:10.1136/archdischild-2019-316951)
Supplement: Supplementary data [file fetalneonatal-2019-316951supp004.pdf]

Appendix 4

Presence of guidelines relating to resuscitation of extremely preterm infants

	No. (%)
Does the institution have relevant local or national professional guidelines relating to resuscitation of extremely preterm infants?	
Yes – The AAP Neonatal Resuscitation Program Guidelines	53 (54)
No	22 (22)
Yes – Local guideline by institution or department	14 (14)
Yes – Philippine Pediatric Society / Philippine Society of Newborn Medicine[a]	7 (7)
Don't know	2 (2)

Abbreviations: AAP – American Academy of Pediatrics

[a] The Philippine Pediatric Society / Philippine Society of Newborn Medicine have not published national guidelines relating to the resuscitation of preterm infants.
