# Supplementary material for: Resuscitation of preterm infants in the Philippines: a national survey of resources and practice
Source: Arch Dis Child Fetal Neonatal Ed. 2019 Jun 14;105(2):209–14. doi: 10.1136/archdischild-2019-316951 (PMC7063403; doi:10.1136/archdischild-2019-316951)
Supplement: Supplementary data [file fetalneonatal-2019-316951supp005.pdf]

Appendix 5

Appendix 5A. Differences in initiation of resuscitation for 23-24 week GA infants by type of institution

	Never/Rarely No. (%)	Often/Always No. (%)	*P* Value[a]
Sector			
Public	25 (86)	4 (14)	.56
Private	40 (80)	10 (20)	
Level			
I / II	21 (84)	4 (16)	>.99
III / IV	42 (81)	10 (19)	
Region			
City	47 (78)	13 (22)	.36
Provincial	13 (93)	1 (7)	
District	5 (100)	0	

[a] Derived using Fisher's Exact test.

Appendix 5B. Reasons why hospitals did not always resuscitate infants at 27-28 weeks GA

	No. (%)[a]
Parent's request to withhold / Financial capacity of family to pay for care	6 (35)
Estimated low chance of survival or clinically deemed non-viable	6 (35)
Birthweight below a certain threshold or presence of IUGR	3 (18)
Uncertainty about GA of infant	1 (6)
Unavailability of surfactant	1 (6)

Abbreviations: GA – Gestational Age. IUGR – Intrauterine Growth Restriction

[a] Respondents could select more than one answer. Respondents who answered that they would 'always' resuscitate (n=79), did not answer this question.
