# Supplementary material for: Resuscitation of preterm infants in the Philippines: a national survey of resources and practice
Source: Arch Dis Child Fetal Neonatal Ed. 2019 Jun 14;105(2):209–14. doi: 10.1136/archdischild-2019-316951 (PMC7063403; doi:10.1136/archdischild-2019-316951)
Supplement: Supplementary data [file fetalneonatal-2019-316951supp006.pdf]

Appendix 6

Factors reported to often or always affect clinicians' decision to limit resuscitation at each institution.

	%[a]
Parents' wishes	73.5
Probability of death	67.4
Clinician's morals	67.3
Risk of poor quality of life	66.3
Financial cost (for family)	62.3
Risk of congenital anomaly	59.2
Health resource allocation	42.9
Emotional burden (for family)	33.7
Clinician's religion	32.7
Fear of litigation for clinician	28.6
Infant pain	19.3

[a] Represents the % of clinicians answering 'always' and 'often' in response to the question of whether these factors influence their decision to limit resuscitation.
