# Supplementary figures and images for: Resuscitation of preterm infants in the Philippines: a national survey of resources and practice
Source: Arch Dis Child Fetal Neonatal Ed. 2019 Jun 14;105(2):209–14. doi: 10.1136/archdischild-2019-316951 (PMC7063403; doi:10.1136/archdischild-2019-316951)

## Appendix 7

### Distribution of birthweight thresholds

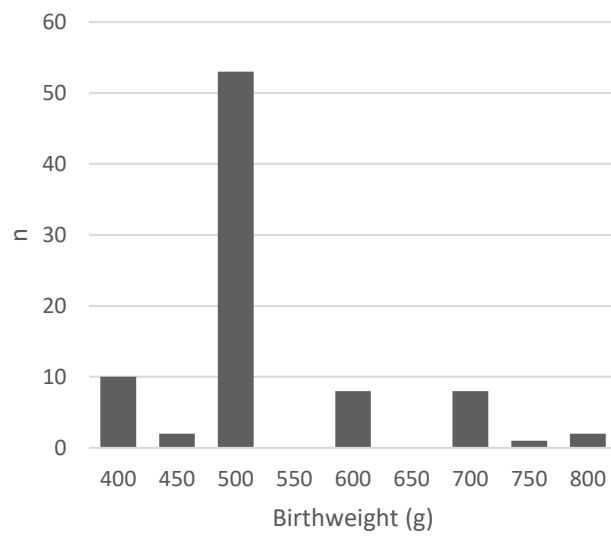

n=84, Median: 500g

Supplement: Supplementary data [file fetalneonatal-2019-316951supp007.pdf]
