# Supplementary material for: Resuscitation of preterm infants in the Philippines: a national survey of resources and practice
Source: Arch Dis Child Fetal Neonatal Ed. 2019 Jun 14;105(2):209–14. doi: 10.1136/archdischild-2019-316951 (PMC7063403; doi:10.1136/archdischild-2019-316951)
Supplement: Supplementary data [file fetalneonatal-2019-316951supp008.pdf]

Resuscitation of Preterm Infants in the Philippines

Oxford Uehiro Centre for Practical Ethics, Littlegate House, St Ebbes St, Oxford OX1 1PT, Tel 01865 286 888

Study title: Resuscitation of Preterm Infants in the Philippines

Outline:

In this study we are asking practicing neonatal specialists from the Philippines about their practice relating to resuscitation of very preterm infants, and the availability of resources in their neonatal units.

The survey will take approximately 20 minutes to complete.

Privacy:

Your answers will be completely anonymous, and we will use all reasonable endeavors to keep them confidential. We cannot trace answers back to you, your computer, or your institution. No identifying details are recorded for either yourself or your institution. IP addresses are not collected. Your answers cannot negatively affect you or your institution in any way.

The questions in the survey may deal with sensitive matters, albeit those which are routinely discussed in newborn intensive care. You may withdraw at any point during the questionnaire for any reason, before submitting your answers, simply by closing the browser. Given anonymity of the responses, you will be unable to withdraw your data once you have completed and submitted the survey.

There will be no direct benefit to you from taking part in the study. It is your choice whether to take part, and you may withdraw from this survey at any point without giving a reason. The data collected however, will help in the construction of locally tailored guidelines for resuscitation of very preterm infants.

Research Team:

- Dean Hayden (BMedSc/MBBS Student) - Monash University / Oxford Uehiro Centre for Practical Ethics
- Prof Dominic Wilkinson - University of Oxford / Oxford Uehiro Centre for Practical Ethics
- Dr Esterlita Uy - Institute of Child Health and Human Development, National Institute of Health / University of the Philippines
- Dr Kat Kat Mendoza
- A/Prof Justin Oakley - Monash Univeristy / Monash Bioethics Centre

The principal researcher is Dean Hayden at the Oxford Uehiro Centre for Practical Ethics in the Department of Philosophy at the University of Oxford.
Email: dbhay3@student.monash.edu

This project has been reviewed by, and received ethics clearance through the University of Oxford Central University Research Ethics Committee CUREC), the Monash University Human Research Ethics Committee (MUHREC) and the University of the Philippines Manila Research Ethics Board (UPMREB). This study has been endorsed by the Philippine Society of Newborn Medicine Board of Trustees under the leadership of Dr. Josie Niu Kho.

Data storage:

Your data will be stored securely online and password-protected with SurveyMonkey and may be used in academic publications. Surveymonkey will store and transfer the information securely with SSL (Secure Sockets Layer: a standard security technology which ensures that all data passed between the web server and browsers remain private and integral) and the data are protected and validated by Norton and TRUSTe. However despite these highly secure measures, please note that as the platform SurveyMonkey is 3rd party, the research team cannot guarantee privacy and confidentiality.

Your IP address will not be collected or stored. All data will be recorded and stored according to the requirements of the Data Protection Act. Only the research team will have access to the project data. The data will be preserved for five years after publication.

This study is in compliance with the Philippine National Health Research System NEGHR guidelines for online/internet research, as well as the Data Privacy Act of 2012 and NEGHR guidelines to online tools for data collections.

What if there is a problem?

If you have a concern about any aspect of this project, please speak to the researcher Dr Esterlita Uy via email at herb_tita@yahoo.com or call +639178980277. Alternatively, you can contact the primary researcher, Dean Hayden at dbhay3@student.monash.edu, who will do their best to answer your query.
The researcher should acknowledge your concern within 10 working days and give you an indication of how they intend to deal with it.
If you remain unhappy or wish to make a formal complaint, please contact the chair of the Research Ethics Committee at the University of Oxford (Chair, Medical Sciences Research Ethics Committee; Email: ethics@medsci.ox.ac.uk; Address: Research Services, University of Oxford, Wellington Square, Oxford OX1 2JD). The chair will seek to resolve the matter in a reasonably expeditious manner.
Alternatively, queries may be directed to the University of the Philippines Manila Research Ethics Board (UPMREB) Chair - Dr Jacinto Blas Mantaring III; Address: UPMREB Panel 1 Chair, 2/f Paz Mendoza, 547 Pedro Gil St, Ermita 1000 Manila; Email: upmreb@post.upm.edu.ph; Tel: +6325264346; Mob: +639273264910.

* 1. If you agree to participate and have read the terms above, please check the relevant box below to get started.

- ◯ Yes, I agree to take part
- ◯ No, I do not wish to take part

* 2. Please certify your age:

- ◯ I am 18 years of age or older
- ◯ I am under the age of 18

Classification of the Hospital

* 3. Which of the following does your hospital fall under?

- ◯ Public / Government Sector
- ◯ Private Sector

Classification of the Hospital

* 4. Which of the following governance organisations does your hospital fall under?

- ◯ Department of Health (Retained Hospital)
- ◯ Local Government Unit

* 5. Regional classification of hospital:

- ◯ City
- ◯ District
- ◯ Provincial

* 6. Which of the following classification levels does your hospital fall under?

- ◯ Level 2
- ◯ Level 3
- ◯ Level 4

Details of the NICU

* 7. Please provide the numbers of births which occurred in your hospital in 2016 (if exact numbers are unavailable, please approximate):

* 8. Please provide the number of preterm births (i.e. <37 weeks gestation) which occurred in your hospital in 2016 (if exact numbers are unavailable, please approximate):

* 9. Number of beds in the NICU:

Official Number:

Maximum (if applicable - i.e. if the number is scaled up in times of need):

Resources in the NICU

* 10. Number of ventilators which are part of the standard NICU setup:

11. How often are **all** mechanical ventilators in use, and **at least one other baby** needs mechanical ventilation?

- Never
- Rarely (less than once a year)
- Some of the time (more than once a year but less than once a month)
- Often (more than once a month but less than once a week)
- Almost always (more than once a week)

Resources in the NICU

* 12. What happens if **all** ventilators are in use and **another baby** needs mechanical ventilation (more than one answer can be selected)?

- [ ] New babies who need treatment are kept comfortable and die.
- [ ] Babies who are currently on the ventilator and who have a low chance of survival are taken off life support.
- [ ] Babies who are currently on the ventilator and who are on low ventilation settings are taken off support in the hope that they won't need it.
- [ ] Hand ventilation (Ambubagging) is attempted if there are individuals able to do so.
- [ ] The family will hire a ventilator.
- [ ] The neonate is transferred to a facility with an available ventilator.
- [ ] Other (please specify)

* 13. How often is surfactant available for preterm infants with respiratory distress?

- ○ Never
- ○ Some of the time
- ○ Only if parents are able to pay for it or find a charity to pay for it
- ○ Always

14. Are there any limits on the use of surfactant? (are there rules on which infants are able to receive surfactant?)

* 15. Approximately how much do families need to pay (if applicable) for the following services? Please provide your answer in Philippines Peso currency:

General NICU Services (per day):

Hiring of a ventilator (per day):

Surfactant administration:

Protocol for Preterm Infants

* 16. Does your institution have relevant local or national professional guidelines relating to resuscitation of extremely preterm infants?

- ◯ No, we do not use a guideline
- ◯ Don't know
- ◯ Yes (please specify)

Protocol for Preterm Infants

* 17. According to the guideline specified in the previous question, what is the **lowest gestational age** at which it would be appropriate to **attempt resuscitation** (including intermittent positive pressure ventilation and intubation)?

Other (please specify):

* 18. According to the guideline specified in the previous question, what is the **highest gestational age** at which it would be appropriate to **not resuscitate** (including intermittent positive pressure ventilation and intubation) if the parents do not wish it provided?

Other (please specify):

Factors in Decision Making

* 19. Do clinicians at your institution make decisions about resuscitating premature infants based on:

- ◯ Gestational age alone
- ◯ Birthweight alone
- ◯ A combination of factors

* 20. For each of the following, please estimate how commonly each factor influences clinicians' decisions to limit resuscitation in your institution:

	Never	Rarely	Sometimes	Often	Always
Risk of congenital anomaly	◯	◯	◯	◯	◯
Risk of poor quality of life	◯	◯	◯	◯	◯
Parent's wishes	◯	◯	◯	◯	◯
Probability of death	◯	◯	◯	◯	◯
Infant pain	◯	◯	◯	◯	◯
Clinician's morals	◯	◯	◯	◯	◯
Clinician's religion	◯	◯	◯	◯	◯
Emotional burden (for family)	◯	◯	◯	◯	◯
Financial cost (for family)	◯	◯	◯	◯	◯
Health resource allocation	◯	◯	◯	◯	◯
Fear of litigation for clinician	◯	◯	◯	◯	◯

Practice of Resuscitation for Preterm Infants

* 21. If a very preterm or extremely preterm infant is born at your hospital, and parents are requesting full active treatment what is the **lowest gestational age** at which you (or your institution) would be prepared to attempt resuscitation (including intermittent positive pressure ventilation and intubation)? (i.e. if the infant is more premature than this you would not resuscitate)

* 22. If a very preterm or extremely preterm infant is born at your hospital, and parents are requesting no active treatment what is the **highest gestational age** at which you (or your institution) would be prepared to **not resuscitate** (including intermittent positive pressure ventilation and intubation), or allow parents to discharge a baby home with the assumption it will die?

* 23. If an extremely low birthweight infant is born at your hospital (precise gestational age unknown), and parents are requesting full active treatment, what is the **lowest birthweight** at which you (or your institution) would be prepared to attempt resuscitation (including intermittent positive pressure ventilation and intubation)? (i.e. if the infant weighs less than this you would not resuscitate).

* 24. For a situation in which the decision to **withdraw** treatment and **palliate** the neonate has been made, how will the cessation of treatment occur?

- [ ] The endotracheal tube will be removed in the unit by doctors or nurses.
- [ ] The endotracheal tube will be removed in the unit by parents.
- [ ] Infant will be Ambubagged to the door of the hospital and parents will remove the endotracheal tube.
- [ ] Infant will not be extubated, but further treatment will not be provided.
- [ ] Other (please specify)

Practice With Regards to Different Gestational Age Bands

The following questions ask you to outline your institutions approach when dealing with neonates born at different gestational ages.

* 25. Would the institution resuscitate?

	Never	Rarely	Sometimes	Often	Always
23-24 weeks	◯	◯	◯	◯	◯
25-26 weeks	◯	◯	◯	◯	◯
27-28 weeks	◯	◯	◯	◯	◯
29-30 weeks	◯	◯	◯	◯	◯
31-32 weeks	◯	◯	◯	◯	◯

Practice With Regards to Different Gestational Age Bands

* 26. For the cases which not all neonates are selected for resuscitation, why are these babies resuscitated and not others?

23-24 weeks

25-26 weeks

27-28 weeks

29-30 weeks

31-32 weeks

* 27. For each of the gestational age bands, what would be your estimate of the chance of survival if the infant is actively resuscitated and taken to the intensive care unit in your hospital?

	0%	1-10%	10-25%	25-50%	50-75%	75-90%	90-99%	100%
23-24 weeks	○	○	○	○	○	○	○	○
25-26 weeks	○	○	○	○	○	○	○	○
27-28 weeks	○	○	○	○	○	○	○	○
29-30 weeks	○	○	○	○	○	○	○	○
31-32 weeks	○	○	○	○	○	○	○	○

Demographic Information

The following questions ask for some demographic information about you, the respondent. They are to help in our interpretation of the survey results.

28. What is you professional role?

- Consultant
- Registrar / Fellow
- Other (please specify)

29. How many years have you worked in newborn intensive care?

30. What is your gender?

- Male
- Female
- Prefer not to say
- Other (please specify)

31. Do you regard yourself as belonging to any religion?

- Yes
- No, I'm an agnostic
- No, I'm an atheist
- Prefer not to say

Demographic Information

32. Which religion do you belong to?

- ○ Christianity – Catholic
- ○ Christianity – Evangelical
- ○ Christianity – Inglesia ni Kristo
- ○ Christianity – Born Again Christians
- ○ Christianity - Other
- ○ Islam
- ○ Buddhism
- ○ Hinduism
- ○ Prefer not to say
- ○ Other (please specify)

33. How important would you say religion is in your life?

- ○ Not important
- ○ Fairly important
- ○ Very important
- ○ Most important

* 34. For each of the following statements, please indicate the extent to which you agree:

	Strongly disagree	Disagree	Neutral	Agree	Strongly Agree
Everything possible should be done to ensure a neonate's survival, no matter however severe the prognosis	○	○	○	○	○
Even with severe physical disability, some life is always better than no life at all	○	○	○	○	○
Even with severe mental disability, some life is always better than no life at all	○	○	○	○	○
The burden that a disabled child will represent for the family is not relevant when making ethical decisions for that neonate	○	○	○	○	○
The high costs of health care for preterm newborns and disabled children should be taken into account when making ethical decisions for a neonate	○	○	○	○	○

THE END

Thanks for your help with this survey!

We really appreciate the time that you have taken to complete it.
